# Pediatric Tinea Capitis: A Retrospective Cohort Study from 2010 to 2021

**DOI:** 10.3390/jof9030366

**Published:** 2023-03-17

**Authors:** Joel Dascalu, Hiba Zaaroura, Yael Renert-Yuval, Ziyad Khamaysi, Emily Avitan-Hersh, Rivka Friedland

**Affiliations:** 1 Department of Dermatology, Rambam Health Care Campus, Haifa 3525408, Israel; joeldascalu@gmail.com (J.D.); zaarourahiba@gmail.com (H.Z.); zkhamaysi@gmail.com (Z.K.); 2 Pediatric Dermatology Unit, Schneider Children’s Medical Center of Israel, Petach Tikva 4920235, Israel; yael.renert@gmail.com; 3 The Ruth and Bruce Rappaport Faculty of Medicine, Technion-Israel Institute of Technology, Haifa 3525422, Israel; 4 Sackler Faculty of Medicine, Tel Aviv University, Tel Aviv 6997801, Israel

**Keywords:** tinea capitis, *Trichophyton tonsurans*, kerion, antifungal treatment

## Abstract

Pediatric tinea capitis displays a wide range of prevalence, with significant variability among populations. We retrospectively extracted the medical records of 456 pediatric patients diagnosed with tinea capitis during the years 2010–2021, from the dermatology outpatient clinics in two tertiary medical centers. Three species were isolated in 90% of patients: *T. tonsurans*, *M. canis*, and *T. violaceum.* While *T. tonsurans* presented a six-fold increase in incidence during the years 2019–2021, *M. canis* maintained stable incidence rates. Furthermore, terbinafine was the most efficient antifungal agent against *T. tonsurans*, achieving complete clinical clearance in 95% of patients, as compared to fluconazole (68%) and griseofulvin (38%) (*p* < 0.001). The mycological cure was recorded in 61/90 (68%) of patients with available data, at an average of 10 weeks. For patients with *M. canis*, griseofulvin and fluconazole were equally efficient (73% and 66%, respectively) (*p* = 0.44). Kerion was described in 36% and 14% of patients with *T. tonsurans* and *M. canis*, respectively, (*p* < 0.001). In conclusion, since 2019, there has been a significant increase in the prevalence of *T. tonsurans*, establishing this pathogen as the most common cause for tinea capitis in our population. Our data suggest that terbinafine is effective and presents high cure rates for tinea capitis in the pediatric population.

## 1. Introduction

Tinea capitis is a common infection of the hair and scalp that occurs primarily in children. Tinea capitis is caused by either anthropophilic, zoophilic, or less commonly, geophilic dermatophytes. Worldwide, *Trichophyton violaceum* and *Trichophyton tonsurans* are the most frequently isolated anthropophilic dermatophytes, while *Microsporum canis* is the most predominant zoophilic dermatophyte [1]. The disease poses a worldwide public health concern due to its great contagious potential, high economic burden, and significant cost of systemic treatments, blood test follow-up, and related adverse events [2]. Additionally, social stigmata may be a major related concern, when children with anthropophilic infection are restricted from attending schools, need to wear hats, or if hair loss and scarring occurs. Overall, children affected by tinea capitis reported a high impact of the disease on their quality of life, correlating with disease severity [3].

Tinea capitis is an extremely contagious infection with known multifactorial risk factors, including overcrowding, poor hygiene, cultural habits, heat, humidity, and socio-economic status. Prevalence rates and culprit organisms widely differ among regions of the world and are related to gender, climate, demographic, and socio-economic factors [2]. For example, in North America, Western Africa, and Eastern Asia, the most common fungi are *T. tonsurans*, *Trichophyton soudanense*, and *T. violaceum*, respectively [1,4]. Tinea capitis also shows variable incidence rates between different ethnicities of the same geographic region [4,5]. However, nation-based studies consistently reveal that males have a higher predisposition to tinea capitis, postulated to be partly related to shorter length of hair [1,4,5]. Worldwide, tinea capitis is more common in populations with poor hygiene and lower socio-economic status [1,2]. For example, children attending a public school are at a higher risk for tinea capitis as compared to children attending a private school [5]. The constant spread of the disease in highly populated areas is also affected by the rates of asymptomatic carriers which constitute a persistent reservoir [6].

Clinical presentations of tinea capitis vary with two major presentations: inflammatory and non-inflammatory. Non-inflammatory tinea capitis presents with mild erythema, broken hairs, and mild scaling. When the infection occurs within the hair shaft, causing an endothrix pattern, the clinical result will be broken hairs manifesting as “black dot” patches. The inflammatory phenotype includes erythematous indurated plaques with pustules, subcutaneous nodules, and sinuses with serous or purulent discharge. This phenotype is usually associated with dermatophytic infections outside the hair shaft (ectothrix). Inflammatory tinea capitis might result in cicatricial alopecia, which is less common in the non-inflammatory phenotype. A severe inflammatory reaction characterized by extensive purulent nodules and sinuses, thick scale, and systemic symptoms including fever and lymphadenopathy is known as kerion celsi. The lymph nodes most commonly involved are the posterior cervical and posterior auricular nodes. These systemic symptoms are helpful diagnostic clinical clues supporting the diagnosis of fungal infection of the scalp over similar dermatologic conditions causing erythema, scaling, or alopecia. A reactive, widespread eczematous rash can also accompany inflammatory tinea capitis. This is referred to as the Id reaction. This inflammatory presentation in both the scalp and the eczematous rash is due to the T-cell-mediated hypersensitivity reaction to the infection by dermatophytes [7]. The inflammatory response is most commonly seen in children ages 5 to 10 and is rare in infancy [8]. While non-inflammatory tinea capitis is usually caused by anthropophilic dermatophytes, inflammatory tinea capitis is usually the result of *M. Canis* infection [9]. 

Another clinical presentation is an asymptomatic carrier state, defined as a condition in which dermatophytes are isolated and identified in a mycological test, with the complete absence of associated clinical signs. The asymptomatic carrier state is most commonly described with anthropophilic species. The prevalence varies in different populations and depends on geographic distribution. For example, in South Africa, *T. violaceum* is endemic and the prevalence of the asymptomatic carrier state reaches up to 49% [1]. In the USA (where *T. tonsurans* is the most common pathogen), the carrier state ranges between 8% to 15% of the general population [1,10]. The asymptomatic carrier state is probably one of the etiologies for the continuous spread of *T. tonsurans* [1].

Although clinical manifestations and epidemiologic data are the basis for suspecting tinea capitis, establishing the diagnosis necessitates a positive result in at least one ancillary test such as direct microscopy of the scale of hair specimen showing fungal hyphae, fungal culture, or molecular biology techniques such as a polymerase chain reaction (PCR) assay aimed at fungal ribosomal DNA genes. Identifying the species of the involved dermatophyte allows the clinician to choose the most suitable treatment [11]. 

Previous reports suggest a high tinea capitis prevalence rate in Israel: the second highest rate in the Middle East [1]. While *M. canis* was the most common pathogen in previous reports, recent reports suggest a pathogenic shift towards *T. tonsurans* [12,13]. The reasons for the increased prevalence of Trichophyton infections are unclear and may be attributed to immigration and globalization [2]. The third most common pathogen, *T. violaceum,* is known as the most prominent pathogen in Ethiopia, where it is found in up to 87% of tinea capitis cases [1]. People of East African descent comprise 1–2% of Israel’s population, originating mainly from Ethiopia. Accordingly, *T. violaceum* was found as the most common pathogen in children of African origin in Israel, while minimal rates of this pathogen were found in other ethnicities [9]. Additional tinea capitis-related pathogens in the United States and France include *Microsporum audouinii* and *Trichophyton soudanense*. The latter were found as the culprit pathogenic fungi among immigrants of African origin [2].

The recognition and awareness of epidemiological trends of tinea capitis and the culprit dermatophytes have an impact on both preventive measures and the selection of antifungal treatment. Therefore, periodical re-assessments are vital to characterize these trends. 

The therapeutic options of tinea capitis vary and include griseofulvin, terbinafine, and fluconazole. In the pediatric population, the treatment of tinea capitis is adjusted by the weight of the patient. Griseofulvin is given at a dosage of 20 mg/kg/day, fluconazole at a dosage of 6 mg/kg/day, and oral granules of terbinafine are given at a daily dosage of 125 mg, 187.5 mg, or 250 mg, adjusted to their weight category of under 25 kg, between 25 and 35 kg, or over 35 kg, respectively [14]. The treatment usually lasts for 6–8 weeks for griseofulvin and fluconazole, and it is shorter for terbinafine (usually 3–4 weeks) [14,15]. Additionally, treatment selection takes into consideration the patient’s age and the relevant pathogen. For example, terbinafine oral granules are authorized from the age of four in North America, but terbinafine is off-label for pediatric tinea capitis in Israel. Griseofulvin is FDA-approved for tinea capitis, while fluconazole is considered an off-label treatment in the USA and Europe [16]. Regarding efficacy, terbinafine was found to be more effective than griseofulvin for tinea capitis due to Trichophyton infections and specifically for *T. tonsurans* infection, while griseofulvin was found to be more effective for tinea capitis caused by *M. canis* [17]. Consideration should also be given to the evolving resistance to griseofulvin [17,18] and to the lack of availability of the drug in some countries [16]. Topical shampoos, including ketoconazole and selenium sulfide, are recommended as adjunctive therapeutic options [19]. As of today, at least in the USA, there are no consensus clinical guidelines for the treatment of pediatric tinea capitis, and a further analysis of tinea capitis management outcomes may contribute to current practices [20]. 

In this study, we examined the prevalence, epidemiology, etiology, and the therapeutic outcomes of pediatric tinea capitis in dermatology outpatient clinics in two tertiary medical centers, as recorded over the past 12 years, delineating the ever-changing pattern of pediatric tinea capitis and its response to treatment.

## 2. Materials and Methods

Included in the study were all consecutive patients aged less than 18 years, who were diagnosed clinically and mycologically to have tinea capitis between 2010 and 2021. We included patients from the outpatient clinics of two tertiary medical centers to better represent the epidemiology of tinea capitis in Israel: the Dermatology Department at Rambam Health Care Campus in northern Israel (70 patients) and Schneider Children’s Medical Center in the center of Israel (386 patients). Two hundred and sixty-six patients were also described in a previous study [12]. 

The tinea capitis diagnosis was confirmed by either culture or a PCR analysis in all cases. The PCR was performed as previously described by Sherman et al. [21]. A commercial kit (Dermadyn IVD dermatophyte detection kit, DYN diagnostics) was used. The kit identifies *Candida albicans*, *Trichophyton interdigitale*, *Trichophyton tonsurans*, *Trichophyton mentagrophytes*, *Trichophyton rubrum*, *Trichophyton soudanense*, *Trichophyton violaceum*, *Trichophyton benhamiae*, *Trichophyton verrucosum*, *Microsporum canis*, *Microsporum audouinii,* and *Epidermophyton floccosum*.

The following data were retrieved from patients’ files: age, gender, ethnicity, residence, clinical manifestations and distribution, microbiological results before and after treatments, treatments (including drug name and dosing regimen), and response. Response to treatment was determined as “complete clinical cure”, “partial response”, and “no response”. We defined the former two terms according to the presence or absence of residual erythema and scale following treatment. The mycological cure was defined as negative mycological testing (either negative culture or PCR analysis). 

The data were transferred to Microsoft Office Excel and subjected to a statistical analysis by the StatPlus statistical package software (StatPlus Prov.7, AnalystSoft Inc., Alexandria, VA, USA). A Mann–Whitney U test was used to compare continuous variables with non-normal distributions. Categorical variables were compared using the chi-square test, with *p* < 0.05 considered significant. NS represented a statistically non-significant result. 

## 3. Results

A total of 456 pediatric patients were identified with a confirmed diagnosis of tinea capitis between January 2010 and December 2021. Demographic characteristics are described in Table 1. Most of the patients in this cohort were aged 4 to 7 years (204, 45%), 69 (15%) were 8 to 10 years old, 106 (23%) patients were 0–3, and 70 (15%) patients were 11 years old or older.

**Figure 1:**
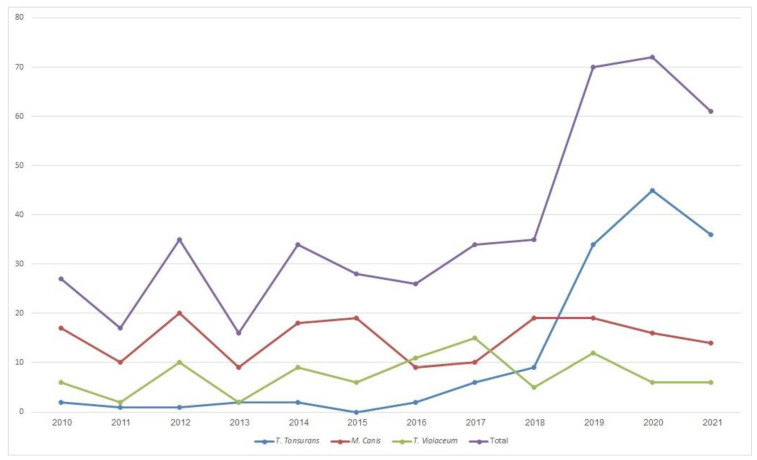
Annual incidence of tinea capitis per species.

The prevalence of each pathogen is described in Table 2. Remarkably, the prevalence of pediatric tinea capitis was stable between 2010 and 2018, but there was a marked increase in the total number of annual cases in the last three years (2019–2021). This rise is consistent with the increased prevalence of *T. Tonsurans* infections, which showed a parallel increasing trend (Figure 1). During this period (2019–2021), *T. tonsurans* was isolated in 57% of all positive microbiological samples, and overall, the majority of *T. tonsurans* cases (115/140, 82%) were diagnosed during these three years. Throughout the cohort, the occurrences of *T. tonsurans* infections were distributed equally among the different seasons, ranging between 23 and 28 cases per season (NS).

We observed a significant male predominance, similar to previous reports. While *M. canis* was detected equally in both genders (39–40% of the cases), *T. tonsurans* was about twice as common among male patients as compared to female patients and accounted for 37% of male tinea capitis and only 18% of tinea capitis in female patients. In contrast, *T. violaceum* showed the opposite trend and was about twice as common in females as compared to males (33% vs. 14%, respectively). 

When comparing the rates of specific pathogens among the different ethnicities (Table 3), we found that *M. Canis* was more common than *T. tonsurans* in the Arab population. Sixty-one patients (58%) of Arab descent were infected by *M. canis* while twenty-three patients (22%) were infected by *T. tonsurans*. The Jewish Caucasians had similar rates of both pathogens, 112 (41%) and 108 (40%), respectively. *T. violaceum* was found in 76% of Ethiopian patients, a significantly higher rate than in patients of either Arab or Caucasian Jewish descent (10% and 8%, respectively).

**Figure 2:**
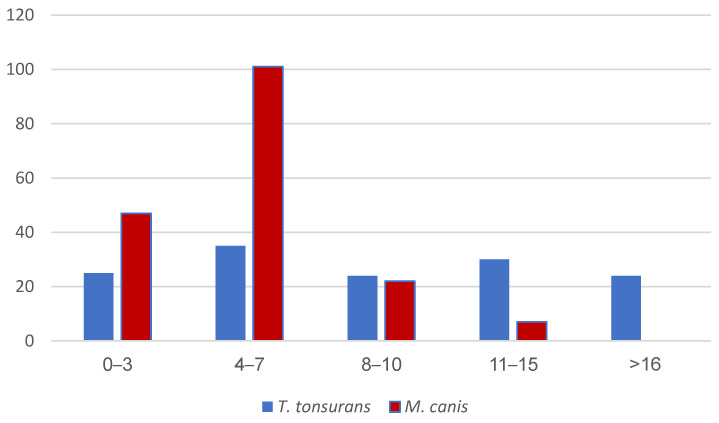
Age distribution of *M. canis* and *T. tonsurans* across study population. *p* < 0.001.

Additionally, we studied the distribution of the pathogens in the different age groups (Figure 2). *M. canis* was more prevalent among the younger patient groups: of one hundred seventy-seven patients infected with *M. canis*, one hundred seventy (96%) were younger than 11 years, and only seven patients (4%) were 11–15 years old. None of the patients were older than 16 years when detected with *M. canis*-related tinea capitis. Conversely, *T. tonsurans* was more equally distributed between the age groups. Of 138 patients, between 25 and 35 patients were infected in each age group. However, when studying the overall tendencies, at the ages of 0–3 years and 4–7 years *M. canis* was more common than *T. tonsurans,* as opposed to similar frequencies for patients aged 8–10 years. In the group of older children aged 11 years or older, the prevalence of *M. canis* markedly decreased and *T. tonsurans* comprised the more common pathogen. Interestingly, in the age group of patients older than 16 years, *T. tonsurans* was the only pathogen causing tinea capitis in non-African patients.

Clinically, the presence of scales was the most commonly reported manifestation (*n* = 425, 96%), followed by alopecia (*n* = 393, 88%), erythema (*n* = 253, 57%), kerion (*n* = 95, 21%), and pustules (*n* = 82, 18%). Kerion celsi and pustules were recorded in 36% and 33% of *T. tonsurans*-infected patients, respectively. *M. canis* was less likely to involve kerion (14%, *p* < 0.001) and pustules (11%, *p* < 0.001). Similarly, *T. violaceum* infections also presented with a lower prevalence of kerion (16%, *p* = 0.001) and pustules (14%, *p* = 0.002) compared to *T. tonsurans* infection.

Data of the applied antifungal agent were available for 424 patients (Table 4). Patients were initially administered with either fluconazole (*n*= 217, 52%), griseofulvin (*n* = 158, 38%), terbinafine (*n* = 41, 10%), or itraconazole (*n* = 4, 1%). About half of the patients adhered to the follow-up recommendations for each treatment; therefore, data of the treatment responses were available for 222 patients (52%). Only four patients were treated with itraconazole and were therefore omitted from the interpretation of the results. Terbinafine showed the highest efficacy for *T. tonsurans*, achieving the best rate of complete clinical cure (19/20, 95%), as compared to fluconazole (30/44, 68%) and griseofulvin (5/13, 38%); *p* < 0.001. *M. canis* and *T. violaceum* also showed high complete response rates of 66–84% to both griseofulvin and fluconazole, with no significant difference between these therapeutics for each pathogen (*p* = 0.44 and *p* = 0.37 for *M. canis* and *T. violaceum*, respectively). 

The overall duration of treatment ranged from 2 weeks to 8 months, with a median of 2 months. The median treatment duration for both fluconazole and griseofulvin was 2 months (ranging from 1 week to 8 months and 1 week to 7 months, respectively). The median treatment duration of terbinafine was 6 weeks (ranging from 1 week to 6 months), which was shorter than griseofulvin (*p* < 0.026) and fluconazole (*p* < 0.111). Only four patients were treated by itraconazole with the median treatment duration of 1 month (3 weeks to 3 months). Culture results following treatment were available for 90 patients, of whom 61 (68%) achieved the mycological cure, at an average of 10 weeks, and 29 patients (32%) required further treatment.

## 4. Discussion

In this study, we presented a cohort of pediatric patients with tinea capitis, over a period of 12 years in two tertiary medical centers located in different districts, and characterized the mycological trends, epidemiology, clinical presentation, and response to treatments. 

Tinea capitis is a global public health issue affecting mostly children, adolescents, and young adults. Although certain pathogens are traditionally related to specific countries, pathogen shifts are described in many developed countries [17,22,23,24,25,26,27,28,29,30,31,32,33,34]. The variability in pathogen distribution necessitates local studies to identify trends and pathogens, and to direct treatment selection. Our large cohort demonstrated a significant increase in *T. tonsurans* infections during the years 2019–2021, which places it as the most common current cause of pediatric tinea capitis. Our previous data in Schneider Children’s Medical Center showed the emergence of a T. tonsurans outbreak in 2019 [12], and similar results were described by Shemer et al. [13]. Thus, the current study both reaffirms the epidemiological trend in an additional tertiary center, and it demonstrates a consistent trend of increased prevalence of T. tonsurans among our patients. Notably, during these years, *M. canis* showed no significant change in prevalence, and was still isolated in a considerable proportion of patients (24% of samples). These results suggest that the rise in *T. tonsurans* infections was not accompanied by a decrease in *M. canis* infection, and that *M. canis* is still a significant pathogen, as in other countries in the Middle East [9]. Therefore, it is possible that the increase in the number of cases of *T. tonsurans* infections in this cohort represents a local increase rather than a pathogen shift. Worldwide, *T. tonsurans* has been associated with several outbreaks in the pediatric population [35]. For example, in a recent single center study in Germany, a ten-fold increase in the number of tinea capitis cases due to *T. tonsurans* was found during 2019–2022 [36]. New emerging pathogens have also been described in other countries around the world, potentially reflecting migrating populations importing certain pathogens to new areas [24,30,31,33,34]. 

The presumed etiology of the significant increase in the prevalence of *T. tonsurans* is unclear. It might be attributed to improper disinfection of barbering tools [13,37,38]. Another explanation could be the rising prevalence of asymptomatic carriers in the household of an infected patient [39,40]. In one study, up to 44% of asymptomatic carriage was noted [39]. These asymptomatic carriers may partially explain the occurrence of infection in younger children who are not exposed to barbering tools. Another possible contributing factor is suboptimal treatment, either due to misdiagnosis, using solely topical agents, inadequate dosage, inadequate treatment duration, or low compliance [41]. Such asymptomatic carriage contributes to the spread of the infection among household members as well as to other families. The equal distribution of *T. tonsurans* incidence among all seasons implies that the rise is probably not a consequence of climate change. Additional studies to determine whether *T. tonsurans* changes its virulence factors and carrier properties are needed. 

*T. violaceum* is known to be the dominant pathogen in North and East Africa [33], but in 2020, *T. tonsurans* was first reported as the predominant agent causing tinea capitis in Ethiopia [42]. The Ethiopian population in Israel accounts for less than 2% of the general population, yet patients of Ethiopian decent represent 16% of this cohort of pediatric tinea capitis [43]. Our study reiterates the high prevalence of *T. violaceum* as the most prominent pathogen in patients of Ethiopian descent (76%), as opposed to other ethnicities (8–10%). It is important to note that although 92% of the Ethiopian children in Israel were born in Israel and this population is well-immersed, we did not see a decrease in the prevalence of *T. violaceum* during the last decade, nor did we see a transition of this pathogen to the non-Ethiopian population [43]. This finding reinforces the hypothesis that some host factors have an impact on the probability of being infected by certain pathogens. 

*M. audouinii*, which was previously reported as an additional major pathogen in African and Ethiopian populations, was rare in our study with only three cases, and none of them were from a person of African descent [44]. These findings raise again the question of whether an infection with a pathogen is exclusively dependent on geographic factors that mainly represent climate and humidity-related influences, or also on genetic predisposition and other host characteristics.

Our study also sheds light on the association of fungal pathogens with kerion celsi. Inflammatory tinea capitis was historically attributed to zoophilic dermatophyte infections [22]; however, during recent decades, *T. tonsurans* was also described as a cause for kerion and severe inflammatory responses [45,46]. In our study, over 40% of patients with tinea capitis due to *T. tonsurans* presented with either pustules or kerion upon physical examination. Interestingly, the prevalence of kerion was lower (14%) in the group of patients diagnosed with *M. canis*. The inflammatory phenotype can also be ascribed to a shift in the virulence of sub-strains of certain pathogens or to a different immunologic response of hosts from different genetic backgrounds, resulting in the varied susceptibility of some patients to developing kerion [47]. 

The age distribution in this cohort demonstrated the well-described epidemiology of tinea capitis with most of the patients being pre-pubertal, i.e., 3–7 years, but tinea capitis was also found in other age groups. This finding solidifies the fact that this cohort was a representative sample, and it allowed us to delve into the distribution of the different pathogens in these age groups. *M. canis* was not isolated in the cultures of patients older than 16 years of age, and it was most prevalent in patients younger than 10 years. Similarly, tinea capitis due to *M. canis* in adolescents is rarely described in the literature [48,49]. One hypothesis for this finding is the fact that infection with *M. canis* requires exposure to an animal reservoir such as cats and dogs, and it is possible that younger children have greater exposure to this potential infectious source. In addition, during puberty there is an increase in sebum excretion on the scalp. The triglycerides in the sebum have a fungistatic effect [50]. This effect might have a greater impact on *M. canis* than on *T. tonsurans.* Factors favoring infection with *T. tonsurans* in children over 10 years old should be further studied. 

The treatment of *T. tonsurans* by terbinafine achieved the highest rate (95%) of complete clinical response, followed by a 68% response rate for fluconazole. Our study replicated encouraging responses for *T. tonsurans* treatment by terbinafine, rendering it as a first choice of treatment for *T. tonsurans* due to its considerably higher efficacy [16].

*T. violaceum* and *M. canis* responded similarly to griseofulvin with complete clinical response rates of 71% and 73%, respectively. These results are aligned with previous data [44]. Nevertheless, there are reports depicting a complete clinical cure for 46% of patients with tinea capitis worldwide [16]. Such a discrepancy might be attributed to the bioavailability of the drug prepared as a syrup in different pharmacies and to the significant influence of concomitant intake. Fluconazole was effective and achieved a clinical cure rate of 68–84% for *M. canis* and Trichophyton species, respectively. Previous studies varied in results, ranging from 40–45% of a complete clinical cure to almost 100% mycological cure [16,51,52]. Overall, we found that griseofulvin did not confer any therapeutic advantage over fluconazole (*p* = 0.44), supporting the use of either drug as a first-line treatment for *M. canis*, and as second-line treatments for *T. tonsurans* infections.

The limitations of this study include its retrospective design and regional relevance. In addition, similar to other tinea capitis studies, the choice of antifungal agent was affected by local availability [16]. Moreover, the microbiology sampling after treatment might have been affected by the level of suspicion of residual disease, and therefore was inconsistent. As our cohort was collected from outpatient dermatology clinics in tertiary hospitals, these patients may represent severe cases of tinea capitis, potentially caused by pathogens associated with more severe clinical phenotypes.

## 5. Conclusions

In conclusion, our study establishes a significant increase in the prevalence of tinea capitis due to *T. tonsurans* since 2019, turning it into the most common pathogen in our population. We also demonstrate that *T. tonsurans* is more common in males and highlight an inflammatory phenotype of this pathogen in a third of pediatric patients. These results emphasize the need to increase awareness of *T. tonsurans* among health professionals in order to efficiently control the spread of this emerging anthropophilic fungus.

Additionally, we show that *M. canis* still constitutes a major cause of tinea capitis, and continues to manifest at a stable prevalence, but is limited to children aged less than 15 years old. We also show the high efficacy of antifungal drugs among the pediatric population, with high rates of both clinical and mycological cures. We suggest that terbinafine should be considered the first choice of treatment for *T. tonsurans* infection, while griseofulvin and fluconazole show similar efficacy towards *M. canis*. 

## Figures and Tables

**Table 1:** Demographic characteristics of study population.

Characteristics	
Mean age (years ± SD)	7.01 ± 4.14
Male (%)	309 (68)
Ethnicity (%)	
Jewish Caucasian	273 (60)
Arab descent	106 (23)
Ethiopian descent	74 (16)
Residence in a rural area (%)	67 (15)
Average time to diagnosis (months ± SD)	3.15 ± 4.8

**Table 2:** Prevalence of main pathogens by gender.

Fungi/Gender	Males	Females	Total Patients
*Trichophyton tonsurans* * (%)	114 (36.9)	26 (17.6)	140 (30.7)
*Microsporum canis* * (%)	121 (39.1)	59 (40.1)	180 (39.5)
*Trichophyton violaceum* * (%)	42 (13.6)	49 (33.3)	91 (20.0)
*Trichophyton mentagrophytes* (%)	9 (2.9)	2 (1.3)	11 (2.4)
*Trichophyton rubrum* (%)	4 (1.2)	1 (0.6)	5 (1.1)
*Nannizzia gypsea* (%)	3 (0.9)	1 (0.6)	4 (0.9)
*Microsporum audouinii* (%)	1 (0.3)	2 (1.3)	3 (0.7)
*Trichophyton verrucosom* (%)	2 (0.6)	1 (0.6)	3 (0.7)
*Trichophyton soudanense* (%)	1 (0.3)	1 (0.6)	2 (0.4)
Unknown (%)	11 (3.6)	6 (4)	17 (3.7)
Total	309	147	456

* Statistical analysis was performed for the 3 most common pathogens (*T. tonsurans*, *M. canis*, and *T. violaceum*), *p*-value < 0.001.

**Table 3:** Prevalence of main pathogens by ethnicity.

Fungi/Ethnicity	Caucasians	Arabs	Ethiopians	All Patients
*T. Tonsurans* * (%)	108 (40)	24 (22)	8 (11)	140
*M. Canis* * (%)	112 (41)	61 (56)	7 (9)	180
*T. Violaceum* * (%)	22 (8)	12 (10)	57 (76)	91
Other fungi (%)	31 (11)	11 (10)	3 (4)	45
Total	273	108	75	456

* Statistical analysis was performed for the 3 most common pathogens (*T. tonsurans*, *M. canis*, and *T. violaceum*), *p*-value < 0.001.

**Table 4:** Patients’ response per fungi to the respective oral regimen.

Fungi	Antifungal Agent	CR (%)	PR (%)	NR (%)	MC (%)
*T. tonsurans* * (*n* = 77)	Griseofulvin (*n* = 13)	5 (38)	4 (31)	4 (31)	3/3 (100)
	Fluconazole (*n* = 44)	30 (68)	9 (20)	5 (11)	12/17 (71)
	Terbinafine (*n* = 20)	19 (95)	1 (5)	0 (0)	5/5 (100)
*M. canis* (*n* = 111)	Griseofulvin (*n* = 48)	35 (73)	4 (8)	9 (19)	17/23 (74)
	Fluconazole (*n* = 59)	39 (66)	9 (15)	11 (19)	11/23 (48)
	Terbinafine (*n* = 4)	0 (0)	1 (25)	3 (75)	0/3 (0)
*T. violaceum* (*n* = 34)	Griseofulvin (*n* = 14)	10 (71)	4 (29)	0 (0)	3/5 (60)
	Fluconazole (*n* = 19)	16 (84)	2 (11)	1 (5)	4/5 (80)
	Terbinafine (*n* = 1)	0 (0)	1 (100)	0 (0)	0

CR: complete response, PR: partial response, NR: no response, MC: mycological cure. * Statistically significant, *p* < 0.001.

## Data Availability

The data presented in this study are available on request from the corresponding author.
